# The Interactions of Obesity, Inflammation and Insulin Resistance in Breast Cancer

**DOI:** 10.3390/cancers7040883

**Published:** 2015-10-26

**Authors:** David P. Rose, Peter J. Gracheck, Linda Vona-Davis

**Affiliations:** 1 Mary Babb Randolph Cancer Center, West Virginia University Health Sciences Center, Morgantown, WV 26506, USA; davidrosemd@hotmail.com (D.P.R.); pjgracheck@mix.wvu.edu (P.J.G.); 2 Department of Surgery, West Virginia University Health Sciences Center, Morgantown, WV 26506, USA

**Keywords:** breast cancer, inflammation, insulin

## Abstract

Obese postmenopausal women have an increased breast cancer risk, the principal mechanism for which is elevated estrogen production by adipose tissue; also, regardless of menstrual status and tumor estrogen dependence, obesity is associated with biologically aggressive breast cancers. Type 2 diabetes has a complex relationship with breast cancer risk and outcome; coexisting obesity may be a major factor, but insulin itself induces adipose aromatase activity and estrogen production and also directly stimulates breast cancer cell growth and invasion. Adipose tissue inflammation occurs frequently in obesity and type 2 diabetes, and proinflammatory cytokines and prostaglandin E2 produced by cyclooxygenase-2 in the associated infiltrating macrophages also induce elevated aromatase expression. In animal models, the same proinflammatory mediators, and the chemokine monocyte chemoattractant protein-1, also stimulate tumor cell proliferation and invasion directly and promote tumor-related angiogenesis. We postulate that chronic adipose tissue inflammation, rather than body mass index-defined obesity *per se*, is associated with an increased risk of type 2 diabetes and postmenopausal estrogen-dependent breast cancer. Also, notably before the menopause, obesity and type 2 diabetes, or perhaps the associated inflammation, promote estrogen-independent, notably triple-negative, breast cancer development, invasion and metastasis by mechanisms that may involve macrophage-secreted cytokines, adipokines and insulin.

## 1. Introduction

Carcinoma of the breast continues to be the most commonly occurring cancer, with the exception of non-melanomatous skin cancers, in American women, although it is second to lung cancer as the most common cause of cancer-related deaths [1]. Similarly, despite the variation in breast cancer incidence rates among women of different European countries, it is the most frequently occurring cancer and also the leading site for cancer-associated deaths [2]. Breast cancer incidence and mortality rates are also increasing rapidly in China, India and other Asian countries, changes in risk that are considered to be a consequence of the adoption of a “western” lifestyle.

Fifty years ago, Fritz de Waard and coworkers proposed that breast cancer arose in premenopausal and postmenopausal women by different mechanisms, the first being related to ovarian factors and the second to obesity and extra-ovarian estrogen production [3]; 10 years later, they reported the results of a prospective study which generated the first evidence that postmenopausal diabetic women are at an increased breast cancer risk, although they considered that the two conditions coexist because of the concomitant high body weight [4].

The seminal studies of de Waard and his colleagues have been confirmed and extended by the progress made in our understanding of adipose tissue and insulin function, recognition of the important role of chronic inflammation in metabolic disorders and carcinogenesis, and advances in steroid biochemistry and tumor biology: these topics form the basis of this review. However, despite these mechanistic advances the situation has also become more acute as a public health issue due to the dramatic increase in the prevalence of obesity, which originated in the industrialized western countries, but has spread throughout the world. Moreover, whereas in the past obesity very largely affected the middle-aged and the elderly, it is now a serious problem in childhood and adolescence [5].

Data for 2005–2006 from the National Health and Nutrition Examination Survey showed that 7.7% of the adult United States population aged 20 years or older had diabetes and another 5.1% were believed to have undiagnosed disease [6]. Type 2 diabetes accounts for 90% to 95% of all diabetes, and is characterized by hyperinsulinemia in the earlier stages of the disease; later insulin deficiency occurs due to β-cell decompensation. There is a positive association between the body mass index (BMI) and the risk of type 2 diabetes. By the criteria established by the World Health Organization, a body mass index (BMI) of 25.0–29.9 kg/m^2^ is regarded as being overweight and values of 30.0 kg/m^2^ or higher as indicative of obesity. When Colditz *et al.* [7] carried out a prospective study of women aged 30–55 years on entry who were followed for eight years, an increased risk of diabetes became evident with BMI levels above 22 kg/m^2^, and for women with a BMI of 33 kg/m^2^ or higher it was estimated that in 98% of cases a diagnosis of diabetes was attributable to the obesity. Consistent with these observations, there is a dramatic and continuing elevation in the prevalence of type 2 diabetes in American children and adolescents which is ascribable, at least in part, to the increasing obesity rate [8].

Because breast cancer, obesity, and type 2 diabetes are all common diseases in Western populations, caution is needed in ascribing causality to associations observed in epidemiological studies, and it is important to demonstrate biological feasibility. On the other hand, their high prevalence also means that large segments of the female population are at risk and so low levels of statistical significance may be biologically important.

One unifying aspect of our review is the role of chronic inflammation in the clinical and biochemical complications of obesity and insulin resistance and in breast cancer causation and biology. Inflammation of the adipose tissue, with infiltration by cytokine and eicosanoid-secreting macrophages, occurs in association with obesity and type 2 diabetes [9], and, as we will examine in some detail, its presence in the breast creates local conditions that are favorable to breast epithelial cell transformation, cancer cell proliferation and invasion, and tumor-related angiogenesis. As the review develops we will suggest that obesity *per se* may not have a direct causal role in breast cancer pathogenesis or expression of a metastatic phenotype, but that this association arises in those obese women with secondary adipose tissue inflammation. This review differs from other reviews on the topic by postulating that metabolically-compromised obesity and adipose tissue inflammation through mechanisms that may involve macrophage-secreted cytokines, adipokines, and insulin play a major role in breast cancer risk, particularly in triple-negative, estrogen-independent breast cancer.

## 2. Adiposity, Inflammation and Type 2 Diabetes

The principal sites of body fat accumulation are the upper abdomen and around the hips and thighs. The ratio of the waist-to-hip circumference (WHR) has been the most frequently used measurement to assess body fat distribution in epidemiological studies, with a high ratio indicating central (visceral or “upper body”) obesity, although more recently it was demonstrated that the waist circumference alone provides an equally good, or even better, assessment of the visceral adipose tissue component [10]. It is the fat that accumulates around the abdominal viscera that is associated with the metabolic complications of obesity, and visceral fat is quantitatively the principal site of adipose tissue inflammation and a major contributor to insulin resistance [11,12]. Although adipocytes comprise the highest percentage of cells within adipose tissue, there is evidence that adipocytes themselves may not contribute proinflammatory cytokines unless there is obesity-associated insulin resistance and inflammation. Obese mice and humans typically have large number of macrophages that comprise up to 40% of resident cells in adipose tissue [13]. They become the main source of proinflammatory cytokines such as tumor necrosis factor-α (TNF-α) and interleukin (IL)-6 [13,14,15,16], and the chemokine monocyte chemoattractant protein-1 (MCP-1) that is elevated in both the adipose tissue [13,14,17] and plasma [17] of mice with diet-induced obesity. The chemokines secreted by adipose tissue macrophages recruit additional leukocytes, so maintaining and expanding the inflammatory process.

Cinti *et al.* [18] originally described the presence of “crown-like structures”, a specific histological feature of adipose tissue inflammation, in increased numbers in obese mice and humans. They are formed by the aggregation of infiltrating macrophages around individual adipocytes, which causes cell death and the formation of a syncytium of lipid-containing giant multinucleated cells, and are particularly prominent in insulin-resistant obese individuals when they are responsible for elevated TNF-α mRNA expression [15]. The pathological abnormalities are accompanied by an increase in serum inflammation biomarkers, including C-reactive protein (CRP), the cytokines IL-1β, IL-6 and TNF-α [19,20,21,22], and MCP-1 [22], although results with TNF-α have been somewhat inconsistent, perhaps because it is expressed locally [15] and exerts its bioactivity primarily by a paracrine mechanism [23].

Macrophages exhibit distinct sets of functions that are determined by local environmental stimuli (Table 1). The “classically-activated” (M1) macrophages are produced from monocytes in response to a combination of interferon-γ and lipo-polysaccharide and secrete high levels of TNF-α and other proinflammatory cytokines, including IL-6 and IL-12, but only low levels of the anti-inflammatory cytokine IL-10 [24]. In contrast, the M2 phenotype has anti-inflammatory properties and has been subdivided into two categories (Table 1). The M2a (“alternatively activated”) macrophages are formed under the influence of IL-10 plus two other anti-inflammatory cytokines, IL-4 and IL-13, and are involved in tissue repair and angiogenesis [25]. The M2b (“regulatory”) macrophages, which have a key role as inhibitors of inflammation by their suppression of inflammatory cytokine expression, produce high levels of IL-10, but, in contrast to MI macrophages, only low levels of IL-12.

**Table 1:** Macrophage phenotypes, their induction, function as a source of cytokines, and bioactivities.

Functions	Macrophage Phenotype		
	M1	M2	
**Phenotypic induction **	Proinflammatory	Anti-inflammatory Anti-insulin resistance IL-10 (as induced by adiponectin), plus IL-4 and 3	
	Pro-insulin resistance		
	INF-γ with LPS		
		M2a “alternatively activated”	M2b “regulatory”
**Cytokines**	TNF-α	IL-4	IL-10
	IL-6		
	IL-12		
	IL-1β		
**Chemokine**	MCP-1 *	MCP-1 *	
**Bioactivity**	Activates NF-κB	Suppresses NF-κB; Pro-collagen synthesis; Anti-inflammation tissue repair	

M1 macrophages secrete high levels of the proinflammatory ILs, but only small amounts of anti-inflammatory IL-10. M2a macrophages secrete IL-4, which stimulates arginase activity essential for polyamine and hence collagen synthesis. M2b macrophages, anti-inflammatory, secrete high levels of IL-10. * MCP-1 overexpression occurs as a component of a mixed M1/M2 phenotype in tumor-associated macrophages. Abbreviations: INF, interferon; LPS, lipo-polysaccharide.

Macrophages play an important role in the development of tumor-related neovascularization. Tumor-associated macrophages have been shown to possess features of the proinflammatory M1 phenotype in the early stages of tumorigenesis, but switch to an M2-like phenotype, with the acquisition of proangiogenic capability including the expression of vascular endothelial growth factor (VEGF) and basic fibroblast growth factor [26] and MCP-1 [27]. Vascular endothelial cells possess MCP-1 receptors, and the chemokine stimulates capillary tube formation *in vitro* and the entire process of neovascularization *in vivo*, activity that is independent of MCP-1 effects on chemotaxis [27]. Macrophages in proximity to a cancer and under the paracrine influence of tumor cell-secreted proteins may actually possess features of both M1 and M2 phenotypic expression. Zinc deficiency has been reported to increase macrophage infiltration in adipose tissue and exacerbate this problem [28].

It is the M1 macrophages that create the proinflammatory environment that blocks the action of insulin on the adipocytes, so promoting the development of insulin resistance and type 2 diabetes, and obesity not only stimulates their infiltration and migration into the adipose tissues, but also induces a redistribution of the macrophage population in favor of the M1 phenotype with overexpression of TNF-α and IL-6 [24,29]. Fujii *et al.* [30] expanded on these observations and showed that when the phenotype present in adipose tissue of mice fed a high-fat diet was manipulated so as to replace M1 with M2 macrophages, there was a loss of proinflammatory cytokines and reversal of the associated insulin resistance.

In some other studies, the serum IL-6 and CRP, but not the TNF-α, concentrations were related to obesity rather than type 2 diabetes [22,31], although results from a prospective health study of middle-aged American women found increasing plasma levels of CRP, IL-6 and MCP-1 to be associated with a higher risk of developing type 2 diabetes, relationships that although attenuated continued to possess a high level of statistical significance after adjustment for the BMI [32].

Adiponectin is a protein that is secreted by mature adipocytes and circulates in the plasma at concentrations that are inversely related to the BMI and are reduced in type 2 diabetes [33]. Whereas obesity favors the production of M1 macrophages [24], which is associated with reduced adiponectin mRNA expression by the infiltrated adipose tissue and elevations in circulating IL-6 and CRP [34], adiponectin promotes a shift to the M2 phenotype and so is an anti-inflammatory adipokine [35]. A different study noted that high plasma concentrations of omega-3 fatty acids were associated with increased adiponectin levels in mice [36].

Recent studies have indicated a role for omega-3 fatty acids and adiponectin production. It was shown that high levels of omega-3 fatty acids prevent abdominal adiposity and the same study also showed that omega-3 fatty acids are inversely correlated with BMI [37]. Interestingly high levels of omega-3 fatty acids have been correlated with high adiponectin levels in patients with type 2 diabetes [38]. This is interesting because it demonstrates a dietary control mechanism for adiponectin in patients who are experiencing type 2 diabetes.

A metabolic connection between type 2 diabetes and postmenopausal breast cancer, and one distinct from any concomitant obesity, was emphasized by epidemiological studies that associated hypoadiponectinemia with both diseases (reviewed by Vona-Davis and Rose, [39,40]). Engeli *et al.* [34] found that the inverse relationships between insulin resistance in non-diabetic postmenopausal women and adiponectin mRNA expression and the plasma concentration of the adipokine were independent of the BMI and percent body fat; moreover, the observed inverse correlation between plasma CRP and adiponectin concentrations persisted after adjustment for obesity-related variables. Kloting *et al.* [41] confirmed this independent association when they showed that in two groups with an equally severe degree of obesity (BMI 45.0 ± 1.3 kg/m^2^), those with insulin resistance had pronounced macrophage infiltration of visceral adipose tissue and elevated serum CRP and reduced serum adiponectin concentrations; a higher serum IL-6 level in the insulin resistant group did not reach statistical significance (*p* = 0.08). Insulin resistance in this situation is a consequence, rather than a cause, of the decreased adiponectin expression in adipose tissue.

Non-alcoholic fatty liver disease is characterized by excessive accumulation of lipids within the parenchymal cells, “hepatic steatosis”, and is a consequence of obesity and the metabolic syndrome [42]. There is chronic inflammation, as indicated by the presence of macrophage infiltration and the formation of crown-like structures within the liver [43], and elevated levels of high-sensitivity CRP [42] and MCP-1 expression in the adipose tissue [44]. As may be expected, this spectrum of pathological changes associated with hepatic steatosis is accompanied by hepatic insulin resistance and an increased risk of type 2 diabetes [44,45].

It is accepted that obesity is not a single entity, and that although commonly associated with insulin resistance and metabolic disorders such as type 2 diabetes and atherosclerotic disease this is not always the case [12,46,47]. Studies from Europe [46] and the United States [47] have indicated that 25%–30% of obese individuals continue to be insulin sensitive; indeed, they maintain metabolic health and do not exhibit the risk factors for cardiovascular disease-hypertension, dyslipidemia, abnormal glucose metabolism-which often accompany obesity [47], and have lower levels of ectopic fat in the liver and skeletal muscle than individuals with insulin resistance [46]. Moreover, metabolically healthy obesity is characterized by lower levels of proinflammatory cytokines and higher serum adiponectin concentrations [48].

The situation was shown to be even more complex by the demonstration that another subset of women exists whose BMI values are in the normal range, but who have a high level of visceral adiposity and are hyperinsulinemic or exhibit insulin resistance [49]. An analysis by Wildman *et al.* [50] of data from 1889 postmenopausal American women who participated in the Women’s Health Initiative Observational Study found that 45.1% of all those of normal weight (BMI < 25 kg/m^2^) had an increased risk of type 2 diabetes and cardiovascular disease as judged by the presence of cardiometabolic abnormalities (“metabolically obese-normal weight”), compared with 72.3% of all overweight or obese women (BMI ≤ 25 kg/m^2^). These women also had plasma biomarkers of adipose tissue inflammation, including elevated CRP and, to a lesser degree, TNF-α and IL-6 concentrations.

## 3. Obesity, Type 2 Diabetes and Breast Cancer

### 3.1. Obesity

Obesity in postmenopausal women is associated largely with estrogen receptor (ER)-positive and progesterone receptor (PR)-positive breast cancers, consistent with the major role played by estrogens synthesized by the stromal cells of adipose tissue in the pathogenesis of these tumors. Epidemiological studies have shown that before the menopause, obesity is either not associated with most molecular subtypes of breast cancer, or, in younger premenopausal women, actually has a protective effect; moreover, the tumors that do develop are less likely to be ER/PR-positive (reviewed by Rose and Vona-Davis, [51]). An important, but not well understood exception is the triple-negative molecular subtype, which is ER and PR-negative and does not express epidermal growth factor-2 (HER-2) receptors; these aggressively metastatic tumors occur predominantly in premenopausal women and constitute approximately 10%–15% of breast cancers in white, but a higher prevalence in black, women. There have been a number of reports that the risk for triple-negative breast cancers is positively associated with obesity, which in a meta-analysis of 11 studies by Pierobon and Frankenfeld [52] was found to be restricted to premenopausal women.

The influence of adiposity on postmenopausal breast cancer risk has been shown to apply specifically to upper body, visceral, obesity as demonstrated by epidemiological studies using the WHR or waist circumference [53,54], a relationship that may be lost in women who are receiving hormone replacement therapy [53].

Pre-existing obesity, a high WHR and postoperative weight gain are also related to a poor prognosis in breast cancer, but in contrast to disease risk there is no modifying influence of menopausal or ER/PR status [55,56]. In postmenopausal breast cancer, elevated estrogen production in the adipose tissue is again a likely major contributor to the adverse effect of excess body fat, as is illustrated by impairment of the clinical response to treatment with aromatase inhibitors [57,58]. However, in premenopausal patients this is an unlikely mechanism because not only is most of the circulating estrogen produced in the ovaries, but the majority of the tumors are estrogen independent, and obesity-related stimulation of breast cancer growth, invasion, and metastasis is derived from non-steroidal factors, including proinflammatory cytokines, leptin, eicosanoids, and insulin [51].

The description of metabolically benign obesity and the recognition that metabolic abnormalities can occur in individuals with adipose tissue inflammation but a normal BMI have important implications for the association between obesity and breast cancer, because they suggest that in the first situation reliance on the BMI to define adiposity may overestimate, and in the second may underestimate, the level of risk.

### 3.2. Hyperinsulinemia

Studies of the relationship between the plasma insulin concentration and breast cancer risk have given conflicting results, and an assessment published by Autier *et al.* [59], which was based on an examination of six published reports, concluded that there is little evidence for an association. However, there are a number of potentially confounding factors that have not always been considered and may obscure a true relationship; these include the influence of menopausal status, hormone replacement, and the interaction with adiposity. For example, one of the studies examined by Autier and coworkers concluded that there was no evidence of an association between plasma insulin concentrations and risk [60], but any influence of hormone replacement therapy on the results is difficult to judge because the available information was insufficient to be sure that all users were excluded. Also, the data were not stratified on the basis of menopausal status, although an analysis based on age below or above 55 years did not show a significant association between quartiles of insulin values and breast cancer risk. Mink *et al.* [61] studied participants aged 45–64 years in the prospective Atherosclerosis Risk in Communities Study, 187 of who had developed breast cancer after an average follow-up period of 7.1 years. There was no demonstrated relationship between breast cancer risk and the fasting serum insulin, but there were only 136 cases aged ≥51 years and no adjustment was made for hormone replacement use.

Overall, it does appear that high circulating insulin concentrations in fasting non-diabetic postmenopausal women who are not taking replacement hormones are positively associated with breast cancer risk [62,63]. One prospective study from the United States that gave a positive result is particularly instructive because the fasting serum insulin concentrations were determined at intervals over the entire observation period [63]. The postmenopausal women with insulin levels in the upper tertile were more than twice as likely to develop breast cancer as those in the lowest tertile. The serum insulin and glucose values were used to calculate the homeostasis model assessment (HOMA)-insulin resistance index, which provides a closer reflection of the insulin resistance as indicated by the euglycemic-hyperinsulinemic clamp method than the fasting plasma insulin alone [64], and this also showed a positive association with postmenopausal breast cancer risk. There is some inconsistency in the reports of studies of breast cancer risk and insulin in premenopausal women, but overall no relationship has been observed with either serum radioimmunoassayable insulin itself, or C-peptide, a subunit of insulin that gives an indication of insulin production and the degree of insulin resistance [40].

There have been few studies of the effect of serum insulin levels on the invasive phenotype in breast cancer, or metastatic behavior and prognosis, although two studies by Goodwin and her colleagues did find that elevated plasma insulin concentrations in both non-diabetic premenopausal and postmenopausal breast cancer patients were associated with an increased risk of recurrence at distant sites and a poor prognosis [65,66]. Also, a nested case-control study by Borugian *et al.* [67] found that when comparing the highest and lowest tertiles, non-fasting serum insulin concentrations showed a trend, although not statistically significant at the 0.05 level, for a positive correlation with breast cancer mortality in postmenopausal (odds ratio, 1.9; 95% confidence interval, CI, 0.7–6.6, p trend = 0.10), but not premenopausal (odds ratio, 0.9; 95% CI, 0.3–2.9, p trend = 0.75) women. Similar results were seen for the serum C-peptide.

The presence of obesity in both premenopausal and postmenopausal breast cancer patients is associated with recognized pathological predictors of a poor prognosis, including large tumor size, metastatic involvement of the axillary lymph nodes, and high histologic grade (reviewed by Rose and Vona-Davis, [68]). Goodwin *et al.* [65] observed a similar relationship between the plasma insulin and tumor stage, nodal involvement and histologic grade, which was independent of accompanying adiposity, in their prospective study of insulin in non-diabetic patients and breast cancer outcome.

### 3.3. Type 2 Diabetes

Larsson *et al.* [69] published a literature review and meta-analysis in 2007 that examined the relationship between diabetes and breast cancer risk; only studies in which the participants were aged 30 years and older when diabetes was diagnosed were included in order to exclude type 1 disease. Fifteen of the 20 eligible studies found that diabetes was associated with an increased breast cancer risk; a meta-analysis of all 20 showed a 20% increase in women with diabetes compared to non-diabetics with a relative risk of 1.20 (95% CI, 1.12–1.28). Three of the publications reported their results after stratification by menopausal status and five others included only or very largely postmenopausal women; analysis of these eight indicated that type 2 diabetes was associated with postmenopausal breast cancer: a meta-analysis by Boyle *et al.* [70] gave similar results, with the risk of postmenopausal breast cancer being increased by 27% in the presence of type 2 diabetes; it was unchanged in premenopausal women. De Bruijn *et al.* [71] also published a meta-analysis, in this case involving studies of various design published since 2007. Diabetes, largely specified as being type 2, was associated with an increased breast cancer risk, with an overall hazard ratio of 1.23 (95% CI, 1.12–1.34).

Type 2 diabetes has also been associated with a poor breast cancer prognosis. In addition to their examination of type 2 diabetes and risk, Larsson *et al.* [69] performed a meta-analysis of five cohort studies and found an overall statistically significant positive relationship between the presence of type 2 diabetes and breast cancer mortality; however, there was a lack of consistency in the results from the individual studies and in several it was not clear that death was specifically due to breast cancer and not another cause of diabetes-related death (“all-cause mortality”). Another meta-analysis, based on six studies none of which were included in the analysis by Larsson *et al.* [69], was reported by Peairs *et al.* [72] and this showed a 49% higher risk of all-cause mortality in patients with pre-existing type 2 diabetes and breast cancer. Only two of the six studies reported breast cancer-specific mortality, but with conflicting results: one found no effect [73]; the other showed an increase, but only in patients who had received chemotherapy [74].

A substantial and detailed comparative study of breast cancer-specific and overall survival by Schrauder *et al.* [75] with 276 patients in the diabetic group and 3780 non-diabetics was published too late for inclusion in the meta-analyses. It found that while overall mortality was higher in the diabetic patients, there were no differences in distant metastasis-free and local recurrence-free survival between the two groups. The meta-analysis-based study reported by De Bruijn *et al.* [71] included a mortality assessment and found that a history of diabetes was associated with an increase in breast cancer-specific mortality. Lastly, Jiralspong *et al.* [76], in 2013, published the results of a study of 6342 patients with stage I to III breast cancer in which, after adjustment for a number of patient- and tumor-related variables, including the BMI, the presence of diabetes was associated with elevated hazard ratios for recurrence-free survival (1.21; 95% CI, 0.98–1.49) and overall survival (1.39; 95% CI, 1.10–1.77); the hazard ratio for breast cancer-specific survival was 1.04 (95% CI, 0.75–1.45).

Women who have been successfully treated for one breast cancer are at a higher risk of a second primary breast cancer compared to the risk of breast cancer for women in the general population and this is increased further by the presence of obesity [77] or type 2 diabetes [78]. In the study by Li *et al.* [78], diabetic women had a 2.2-fold increased risk of primary contralateral breast cancer, which was substantially greater in women diagnosed with their first ER-positive cancer before they were 60 years of age than those aged 60 years or older; there was no interaction with the BMI.

Overall, the published studies support the view that hyperinsulinemia and type 2 diabetes are both causally related to breast cancer risk, but that, as in the case of obesity, the association is largely restricted to postmenopausal women. The influence of type 2 diabetes on breast cancer prognosis requires further examination so as to provide clarification of its effect on breast cancer-specific mortality. Also, given the therapeutic implications, potential interactions between hyperinsulinemia, type 2 diabetes and estrogen-dependence need further investigation in the clinical setting, as does the potential for confounding by deviation from the optimal protocols for cytotoxic chemotherapy in diabetic breast cancer patients [79].

A number of reports have described more advanced disease in type 2 diabetic patients with newly diagnosed breast cancer compared with non-diabetic controls, although despite the consistent difference in the prevalence of metastasis to the axillary lymph nodes shown in Table 2, for two [80,81] of the six studies the trend for larger primary tumors in the patients with diabetes was not statistically significant. Hou *et al.* [82] were the only investigators to assess tumor cell proliferation, and found it to be *reduced* in diabetic patients, as judged by the expression of Ki 67, a nuclear proliferative biomarker that has been associated with a poor prognosis [83], and is elevated in breast cancers from obese patients [84]. This is an unexpected result given that, as discussed later, insulin is a mitogen for breast cancer epithelial cells. However, it may be relevant that in a preclinical study, Hadad *et al.* [85] found that metformin, a biguanide hypoglycemic agent prescribed extensively in the treatment of type 2 diabetes, reduced breast cancer cell Ki 67 expression and so its use may have had a confounding effect.

**Table 2:** Tumor size and lymph node involvement in non-diabetic and diabetic breast cancer patients.

Reference (No.)	T1		T2		T3 + T4		Positive Lymph Nodes		BMI	
	ND %	D %	ND %	D %	ND %	D %	ND %	D %	ND	D
[73]	67.8	62.8	26.4	30.9	4.8	5.3*f*	22.1	24.8*f*	not given	
[74]	57.9	48.0	35.0	41.2	7.1	10.8 **	34.9	41.1 *	25.9 ± 4.7	28.8 ± 5.6*f*
[79]	55.5	33.8	37.3	55.4	7.2	10.8 *	24.0	33.0	26.9 ± 4.1	29.7 ± 4.5*f*
[80]	34.3	25.9	50.3	55.9	15.4	18.2	43.4	56.6*f*	26.9 ± 4.0	27.1 ± 3.8
[81]	18.0	10.5	60.5	62.9	21.5	26.7	53.0	66.0 *	not given	
[82]	35.5	28.5	52.2	55.2	12.3	16.3*f*	43.3	47.5 *	28.1%	47.5%*f*

ND, non-diabetic; D, diabetic. Tumor size: T1, <2 cm; T2, 2–5 cm; T3 + T4, >5 cm. Significantly different from non-diabetics: *f* *p* < 0.001; ** *p* < 0.01; * *p* < 0.05. Hou *et al* [82] reported normal BMI, overweight and obese categories; *p* for trend was significantly higher in the diabetic women (*p* < 0.001).

### 3.4. Obesity-Insulin Resistance Interaction and Breast Cancer

The prominence of central obesity in type 2 diabetes, its association with hyperinsulinemia in non-diabetic women, and its function as a risk factor for postmenopausal breast cancer and negative influence on prognosis, all raise the question of the extent to which the perceived role of hyperinsulinemia is due to the accompanying obesity-related elevation in extraglandular estrogen production that occurs after the menopause.

Some of the studies described in the previous section adjusted their statistical analyses for the BMI. This was done in the meta-analysis of type 2 diabetes and breast cancer risk performed by Larsson *et al.* [69], when it had no significant effect: without adjustment the relative risk associated with the presence of diabetes was 1.19 (95% CI, 1.09–1.30), and with adjustment it was 1.20 (95% CI, 1.09–1.33). Michels *et al.* [86], in an analysis of data from the Nurses Health Study, found that the association of breast cancer risk with type 2 diabetes, which in their study was largely restricted to ER-positive tumors, was not altered significantly by the BMI. However, a later review and meta-analysis of 39 independent risk estimates by Boyle *et al.* [70] showed that the 27% increase in risk (summary relative risk 1.33; 95% CI, 1.18–1.51) associated with type 2 diabetes was reduced to 16% (summary relative risk 1.16; CI, 1.08–1.24) when adjusted for the BMI. Also, epidemiological investigations of risk do have the potential for residual confounding and so it is reassuring that in the prospective study reported by Kabat *et al.* [63] the positive relationship between serum insulin and breast cancer risk was evident in lean postmenopausal women.

Asian-American women are of particular interest in this context because their body size is typically smaller than that of European-American women, and their risk of developing hyperinsulinemia and type 2 diabetes occurs in the presence of relatively low BMI values. Furthermore, a meta-analysis of premenopausal breast cancer risk, the BMI and ethnicity, which confirmed the inverse relationship in white women, showed a positive association for Asian women [87]. A case-control study performed in California by Wu *et al.* [88] found a significant increase in breast cancer risk in women of Chinese, Japanese or Filipino origin with a history of type 2 diabetes, and this was reduced only marginally by adjustment for the BMI or WHR. Moreover, the adverse effect of diabetes on risk was seen in women with a BMI below 22.7 kg/m^2^ (odds ratio = 3.50, *p* = 0.011) rather than those with a higher BMI (odds ratio = 1.39, *p* = 0.23), a cut-off that is lower than that likely to be required to account for any ethnic difference in the definition of overweight/obesity [89]. In this context, studies of adipose tissue inflammation as a BMI-independent risk factor for breast cancer in Asian women would be of interest.

The BMI was reported in four of the six studies of primary breast cancer size and axillary lymph node metastasis summarized in Table 2 and in three of the studies, adiposity was more prevalent in the diabetic patients. Wolf *et al.* [79] calculated the odds ratios to compare the breast cancer pathology with adjustment for the BMI and found that the disease stage was higher and primary tumor size greater in the diabetic compared with non-diabetic women. None of the other three groups of investigators included adjustment for the elevated BMI in their statistical analyses, but Hou *et al.* [82] found no effect of obesity on overall survival in their study.

## 4. Estrogen and Insulin Mechanisms

This discussion of the mechanisms by which obesity and type 2 diabetes promote breast cancer risk and progression focuses on estrogen production and bioactivity and the direct and indirect actions of insulin. Later, consideration will be given specifically to the influence of adipose tissue inflammation on estrogen biosynthesis, and hence specifically on ER-positive breast cancers, and the additional non-estrogenic actions of the inflammatory cytokines, chemokines and leptin that promote breast cancer cell proliferation, invasion and metastasis regardless of ER status.

After the menopause, estrogens are produced almost exclusively by the enzymatic conversion of androstenedione, a C19 steroid secreted largely by the adrenal glands, to the estrogenic C18 steroid estrone; the responsible aromatase system is located in the adipose stromal cells. Obese women possess elevated aromatase activity and in consequence produce increased levels of estrogens, which in postmenopausal women are reflected in relatively high plasma levels and are associated with an increase in breast cancer risk [90,91]. Before the menopause, when the ovaries are the principal source of the plasma estrogens, obesity-induced ovarian dysfunction may be responsible, at least in part, for a reduction in breast cancer risk, although this is an overly simplistic explanation for the adiposity-related differences in menopausal status and breast cancer risk [92].

The subcutaneous and visceral adipose tissue mass provides the principal source of the circulating estrogens that are available for endocrine action on premalignant and neoplastic breast epithelial cells, but in addition, and perhaps more importantly, the breast adipose stromal cells produce a locally high concentration of estrogen which can act on the target tissue by a paracrine mechanism [93]. The level of aromatase activity is a function not only of the total adipose tissue mass, but also, and of particular significance in the present context, the extent of enzyme induction per unit cell by obesity-related mediators, including insulin [94]. Aromatase is the rate-limiting enzyme for estrogen synthesis, but adipose tissue also contains the 17β-hydroxysteroid dehydrogenase which is responsible for the conversion of estrone to the more biologically potent estradiol.

Postmenopausal women with type 2 diabetes frequently have an increase in the plasma estrogens that is distinct from any coexisting adiposity and is presumably a consequence of aromatase induction by insulin [95,96], although this has not been demonstrated directly and it needs to be pointed out that in a study performed by Kalyani *et al.* [96] some effect persisted after adjustment not only for the BMI, but also for insulin resistance as estimated by the HOMA model.

In addition to the increase in extraglandular estrogen biosynthesis, both obesity and type 2 diabetes are associated with an elevation in the level of bioactive estradiol. In healthy individuals approximately 30%–50% of the plasma estradiol is tightly bound to the hepatic protein sex hormone-binding globulin (SHBG), which renders it biologically inert. The plasma SHBG concentration is reduced in obese women and constitutes a further marker of increased postmenopausal breast cancer risk due to the consequent elevation in the level of biologically available hormone [90,91]. The plasma SHBG is also reduced in women with hyperinsulinemia, a relationship which is due to a direct suppressive effect of insulin on SHBG production by the liver, is independent of the BMI [97], and, like the accompanying increase in the bioactive estradiol, is a biomarker of high type 2 diabetes risk [98].

As well as being prerequisites for hormone responsiveness, breast cancer ER and PR expression provide an indication of clinical outcome, receptor negative tumors being predictive of a poor prognosis [99]. Consistent with the reports that type 2 diabetes is associated with reduced breast cancer survival, Srokowski *et al.* [74] found that the prevalence of ER-negative breast cancer was higher in their diabetic patients compared with the non-diabetic group; however, although the small difference, 32.2% *versus* 30.6%, was statistically highly significant (*p* < 0.001), this may have been affected beyond its biological influence by the large sample sizes. In none of the other studies summarized in Table 2 was there a difference in the prevalence of ER-negative tumors, and Michels *et al.* [86] noted that the presence of diabetes in postmenopausal women was actually associated with ER-*positive* breast cancer, as it is in obesity. Schrauder *et al.* [75] found that in their series of patients there was an interaction between diabetes and the risk of distant metastases that was limited to those with ER-negative tumors. Distant metastasis was present in 21% of the diabetic group with ER-negative breast cancers, but only 12.4% of non-diabetics with ER-negative tumors (hazard ratio, HR, 2.28; 95% CI, 1.31–3.97: *p* < 0.01), whereas there was no similar difference for ER-positive tumors (HR, 0.72; 95% CI, 0.41–1.27).

**Figure 1:**
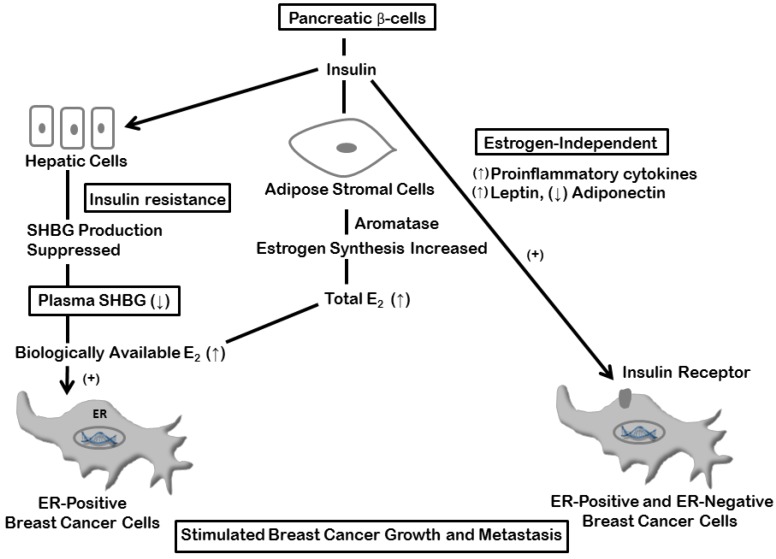
Stimulation of breast cancer cell proliferation and invasion by insulin. Enhanced adipose stromal cell estrogen production and suppression of hepatic SHBG synthesis with elevated estrogen bioactivity stimulate ER-positive cells indirectly by a combination of endocrine and paracrine activities, and insulin action on insulin receptor-expressing cells promotes both ER-positive and ER-negative breast cancer progression. Abbreviations: A2, androstenedione; E1, estrone; E2, estradiol; HSD, 17β-hydroxysteroid dehydrogenase; SHBG, sex hormone-binding globulin.

Four of the six studies in Table 2 provided results for breast cancer PR expression, and in two [80,82], both from China, this was 10%–20% lower in the diabetic groups (*p* < 0.001). Liao *et al.* [80] also found that the difference was confined to tumors from premenopausal patients, although the comparisons were based on small numbers. The study by Hou *et al.* [82], which included 1013 diabetic and 4621 non-diabetic patients, confirmed the observation by Liao *et al.* [80] that the diagnosis of diabetes was associated with PR-negative breast cancer. Berstein *et al.* [100] reported that diabetes is associated with ER-positive/PR-negative tumors in premenopausal women, and Larsson *et al.* [101] found the same relationship with high dietary carbohydrate intake, the glycemic index, and, most strongly, the glycemic load.

Breast cancer epithelial cells possess insulin receptors, and experiments with human breast cancer cell lines have demonstrated that the hormone has direct mitogenic activity which is mediated via the phosphatidylinositol-3 kinase and mitogen-activated protein kinase/Akt signaling pathways; insulin is also a cell survival (anti-apoptotic) agent and enhances tumor cell migration and invasive capacity (reviewed by Rose and Vona-Davis, [102]).

The estrogen-mediated and direct effects of insulin on breast cancer cell proliferation and invasion are summarized in Figure 1.

## 5. Inflammation and Breast Cancer: Estrogen-dependent Mechanisms

**Figure 2:**
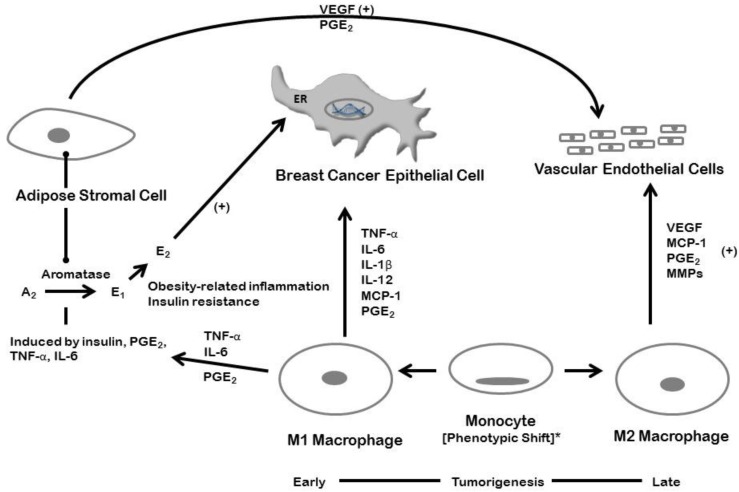
Inflammation and breast cancer: the paracrine interactions of adipose stromal cells and M1 macrophages with breast cancer epithelial cells, and promotion of tumor-related angiogenesis by stromal cell and M2 macrophage-secreted angiogenic factors. * A partial phenotypic shift in favor of M2 macrophages, VEGF production, and angiogenesis may occur later in the course of tumorigenesis. Abbreviations: ER, estrogen receptor; TNF-α, tumor necrosis factor-α; IL, interleukin; MCP-1, monocyte chemoattractant protein-1; PGE2, prostaglandin E2; VEGF, vascular endothelial growth factor; MMPs, matrix metalloproteinases.

The breast adipose tissue can be involved in obesity-related chronic inflammation, with crown-like structure formation and increased cytokine production by the associated macrophages [103,104]. In addition to the endocrine effect of insulin in stimulating aromatase activity [98], TNF-α and IL-6 [105,106], and prostaglandin E2 (PGE_2_) produced under the influence of elevated cyclooxygenase-2 (COX-2) activity [106,107], can all induce the enzyme by paracrine interactions between macrophages and adipose stromal cells, with consequent stimulation of ER-positive breast epithelial cell growth by the locally produced estrogen (Figure 2). These activities within the breast are not necessarily indicated by an increase in the circulating cytokine concentrations.

Menopause is associated with a diet-independent increase in adiposity and insulin resistance. This is modeled by bilateral ovariectomy in mice, which causes body weight gain due to expansion of the adipose tissue mass in perigonadal and inguinal deposits, and infiltration by M1 macrophages, the formation of crown-like structures, elevated levels of TNF-α, IL-1β and IL-6 expression, and insulin resistance [108]. Subbaramaiah *et al.* [109] showed that there was an increase in the number of crown-like structures in the stromal compartment of both the visceral fat and the mammary tissue of high-fat-fed obese mice, together with elevated expression of COX-2 and aromatase. In these experiments, groups of mice were fed a high-fat diet and/or ovariectomized to induce weight gain; the body weights and number of crown-like structures were significantly greater in the ovariectomized mice fed the high-fat diet. The levels of TNF-α, IL-1β and PGE_2_ were also higher in the macrophage-rich stromal-vascular fraction isolated from the mammary glands of the obese mice compared with the same cellular fraction from lean mice; again, the highest levels were present in the combined high-fat-ovariectomized group.

In the context of this review, it is particularly noteworthy that Morris *et al.* [104], in their parallel study of obese postmenopausal women, found that both aromatase mRNA expression and the number of crown-like structures were more closely related to the severity of the inflammation rather than the degree of adiposity. Insulin resistance was not assessed, but, as the investigators pointed out, the observation is consistent with the concepts of metabolically obesity/normal weight and “metabolically healthy obesity”. Moreover, the associated biochemical changes suggest that obesity and type 2 diabetes may promote breast cancer growth and progression, with a major role for aromatase-mediated extra-ovarian estrogen production, in ER-positive, largely postmenopausal, tumors, but estrogen-independent proinflammatory cytokine, adipokine and insulin effects in some ER-negative, predominantly premenopausal, breast cancers.

Cross-talk occurs between ER and NF-κB in breast cancer which is mediated by estradiol and TNF-α; the combined activity of the estrogen and inflammatory cytokine promotes the transition of breast cancer cells to a more aggressive, ER-positive but antiestrogen-resistant phenotype with constitutive activation of NF-κB and an increased propensity for distant metastasis [110]. This role played by NF-κB in promoting aggressive behavior by ER-positive tumors is actually a special case; in general, expression and activation of this transcription factor is associated with high proliferation rates, high histologic grade and invasiveness in ER-negative breast cancer [111].

## 6. Inflammation and Breast Cancer: Estrogen-Independent Mechanisms

In addition to its hormonal effects, dietary-induced chronic inflammation in breast adipose tissue produces a local microenvironment that, regardless of ER status, is conducive to increased tumor cell proliferation and metastatic capacity, and enhanced tumor-related angiogenesis. Kim *et al.* [112] used the obesity-resistant BALB/c strain of female mice to demonstrate that feeding a high-fat diet could stimulate growth of an ER-negative murine mammary carcinoma cell line, and its metastasis from the orthotopic injection site to the lungs and liver, in the absence of excessive body weight gain. This accelerated cancer progression was accompanied by enhanced tumor-related angiogenesis, with overexpression of VEGF, and increased serum concentrations of several proinflammatory cytokines, including IL-6, and leptin.

Transgenic mice that express the polyoma virus middle T oncoprotein (PyMT) provide an alternative model, and one that represents all the stages of breast cancer development. These animals consistently develop mammary lesions that pass through preliminary stages of epithelial hyperplasia and carcinoma *in situ* to produce locally invasive, systemically metastatic, ER-negative cancers. Berryhill *et al.* [113] showed that feeding a diet supplemented with the *trans*-10, *cis*-12 isomer of conjugated linoleic acid (CLA) to ovariectomized PyMT mice increased mammary ductal hyperplasia by a mechanism that was independent of both ovarian and extraglandular estrogen activity, but was causally associated with hyperinsulinemia. The involvement of adipose tissue inflammation was suggested by a previous report by Poirier *et al.* [114] that feeding 10,12 CLA to mice causes increased macrophage infiltration and induction of elevated TNF-α and IL-6 expression.

These mice also have a genetic background that renders them relatively resistant to excessive body weight gain, and in a later study we found that they did not become overtly obese when fed a calorie-rich diet with a high saturated fat content, a situation that, as in the experiment with the murine mammary carcinoma cell line, mimicked the human condition of “metabolically obese-normal weight”. The high fat-fed PyMT mice showed a stimulated primary tumor growth rate and enhanced metastasis to the lungs, effects that were associated with hepatic steatosis, pronounced macrophage infiltration and crown-like structure formation, neovascularization in the peritumoral adipose tissue, and elevated plasma MCP-1 concentrations and adipose tissue MCP-1 expression [115]. A study recently conducted revealed that human COX-2 expression in mice that do not express the gene protects against adiposity, and insulin resistance through induction of genes involved in beta-oxidation, lipogenesis, and lipolysis [116]. Also it was recently shown that autophagy was upregulated in patients with type 2 diabetes in their visceral adipose tissue and could explain why patients with type 2 diabetes have increased inflammation [117].

The synthesis of TNF-α by infiltrating M1 macrophages was found to be enhanced by a paracrine interchange with breast cancer epithelial cells [118], and Hagemann *et al.* [119] showed that culture of an ER-positive human breast cancer cell line with TNF-α-secreting macrophages increased its otherwise poor invasive capacity, an effect that was associated with elevation in the production of matrix metalloproteinases (MMPs), critical for the invasion cascade, and was dependent on increased NF-κB activation. The induction of enhanced MMP-9 expression is also essential for TNF-α-stimulated invasion of triple-negative MDA-MB-231 breast cancer cells [120]. Interleukin-6 is produced by both inflammation-associated macrophages and breast cancer cell-associated fibroblasts, and the stimulation of both ER-positive and ER (triple)-negative breast cancer cell invasion by co-culture with adipose stromal cells was shown to be mediated by IL-6 [121].

In women with breast cancer, inflammation in the tumor microenvironment, with local elevation in the expression of proinflammatory cytokines, is also associated with increased invasiveness and a poor prognosis [122], although the relationship may be influenced by the molecular subtype. Thus, Herrera *et al.* [123] found that the plasma TNF-α concentrations were high in patients with luminal (ER and PR positive, HER-2 negative) tumors, but low in triple-negative breast cancer. However, as described earlier, obesity is a risk factor for triple-negative breast cancer in premenopausal women and was found by Turkoz *et al.* [124] to be associated with reduced overall and disease-free survival; also, this phenotype does exhibit a high degree of NF-κB activation [125] and expression of inflammation-related genes in the surrounding non-tumor cell population [126,127]. Lastly, although further investigation is needed, both diabetes [128] and hyperglycemia when a component of the metabolic syndrome [129] has been identified as adiposity-independent risk factors for triple-negative breast cancer.

The production of proangiogenic proteins such as VEGF, MCP-1, leptin, and TNF-α, by macrophages and adipocytes [26,27,34] and the increased vascular network demonstrated in adipose tissue surrounding experimental mammary tumors is consistent with the described interactions between obesity, inflammation and insulin resistance in breast cancer development. New blood vessel formation occurs in support of adipogenesis, expansion of the adipose tissue mass, and the development of obesity; it also contributes to type 2 diabetes and some of its vascular complications [51,130]. Angiogenesis is also an essential component of human breast carcinogenesis and its progression to metastatic disease, so that its presence at a high activity level is predictive of a poor prognosis [131].

The complex interactions between adipose stromal cells, macrophages, and the epithelial tumor cells in promoting breast cancer growth and invasion, and the associated formation of a new supporting vasculature are summarized in Figure 2.

## 7. Conclusions

There is general agreement that obesity is a risk factor for postmenopausal breast cancer, but the concepts of “metabolically benign obesity” and “metabolically obese-normal weight” developed from studies of type 2 diabetes and atherosclerotic heart disease suggest a new approach to understanding the associations between adiposity and breast cancer. Future research should focus on the relationships between metabolically compromised obesity and adipose tissue inflammation to determine the level of breast cancer risk beyond the limits set by a BMI-based definition of adiposity. Moreover, as illustrated by the special case of triple-negative, hormone-independent, breast cancer in premenopausal women, prospective epidemiological studies should be of sufficient size to permit statistical analysis based on molecular subtyping.

The need for controlled dietary intervention and physical exercise clinical trials to demonstrate reduced breast cancer risk and improved prognosis in obese women may be considered unnecessary, given the accumulated health benefits, although their feasibility has been demonstrated by the successful completion of the Women’s Health Initiative and Women’s Intervention Nutrition studies in the United States. Certainly, biological rationale is provided by the reported effects of body weight reduction on the plasma and adipose tissue concentrations of estrogens and inflammatory biomarkers such as CRP, IL-6 and TNF-α, and a reduction in macrophage number and shift to the anti-inflammatory M2 phenotype in adipose tissue after bypass surgery-induced weight loss [132].

Adipose tissue inflammation occurs in association with type 2 diabetes, but the close correlation with obesity complicates the interpretation of epidemiological studies of this metabolic disorder in breast cancer, which may be confounded by inadequate statistical control for the BMI. Furthermore, biochemical abnormalities such as elevated estrogen bioactivity, reduced circulating adiponectin concentrations, and hyperinsulinemia, which have been identified as contributing factors in breast cancer pathogenesis and progression, occur in both type 2 diabetes and obesity. Nevertheless, review of the epidemiological evidence, supported by laboratory studies that provide biological plausibility [102], lead us to the conclusion that type 2 diabetes does have its own distinct role in breast cancer etiology.

A specific causal relationship between hyperinsulinemia and breast cancer risk and progression is also supported by reports that metformin, which reverses hyperinsulinemia and insulin resistance, is associated with reduced breast cancer risk and improves the response to adjuvant chemotherapy and disease outcome [40]. Metformin has also been shown to inhibit Src-mediated induction of the inflammatory pathway, with suppression of IL-6 expression and NF-κB activation, perhaps due to blocking a transduction pathway linked to glucose and anabolic metabolism [133].
